# Supplementary figures and images for: Nutrient limitation of algae and macrophytes in streams: Integrating laboratory bioassays, field experiments, and field data
Source: PLoS One. 2021 Jun 18;16(6):e0252904. doi: 10.1371/journal.pone.0252904 (PMC8213151; doi:10.1371/journal.pone.0252904)

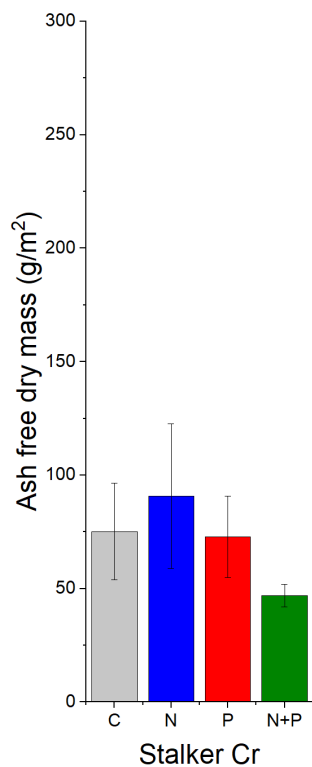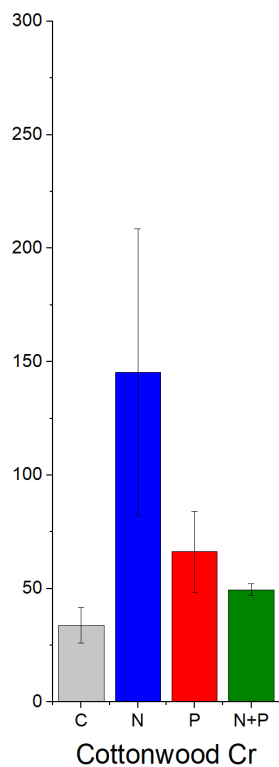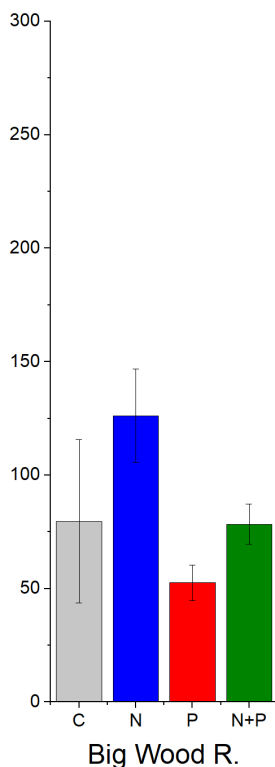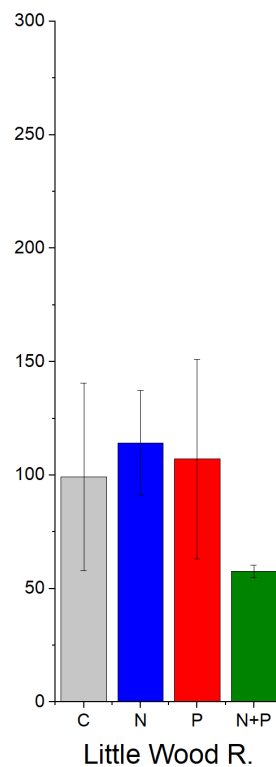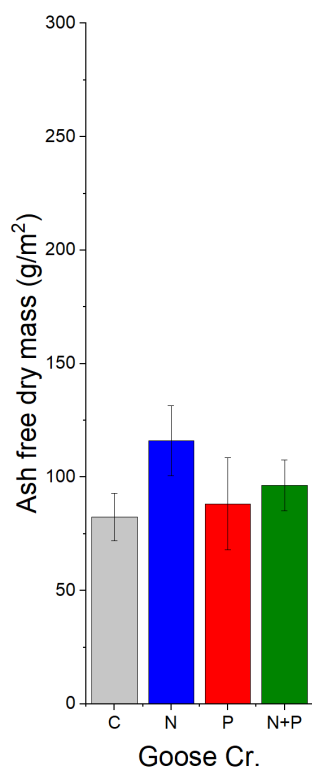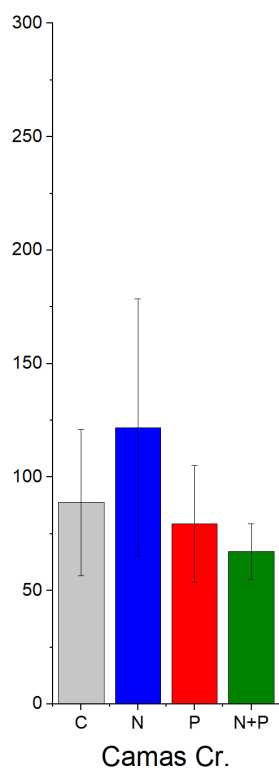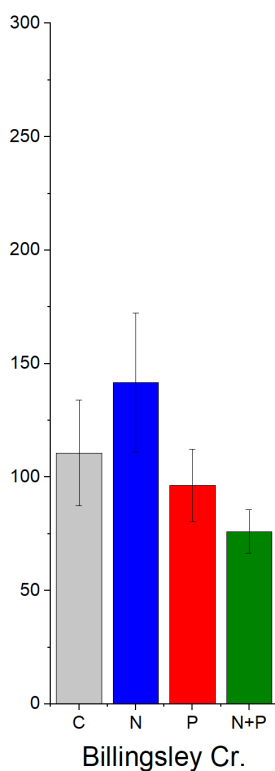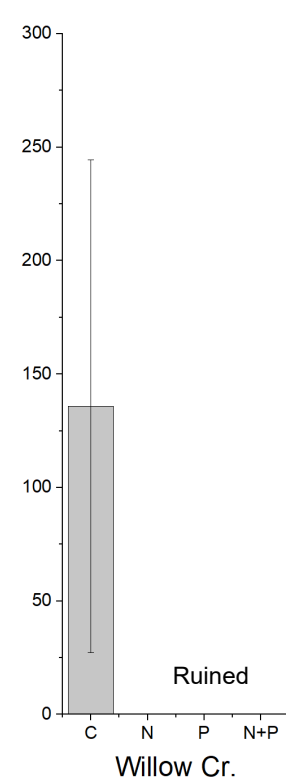

Supplement: S1 Fig — (PDF) [file pone.0252904.s001.pdf]

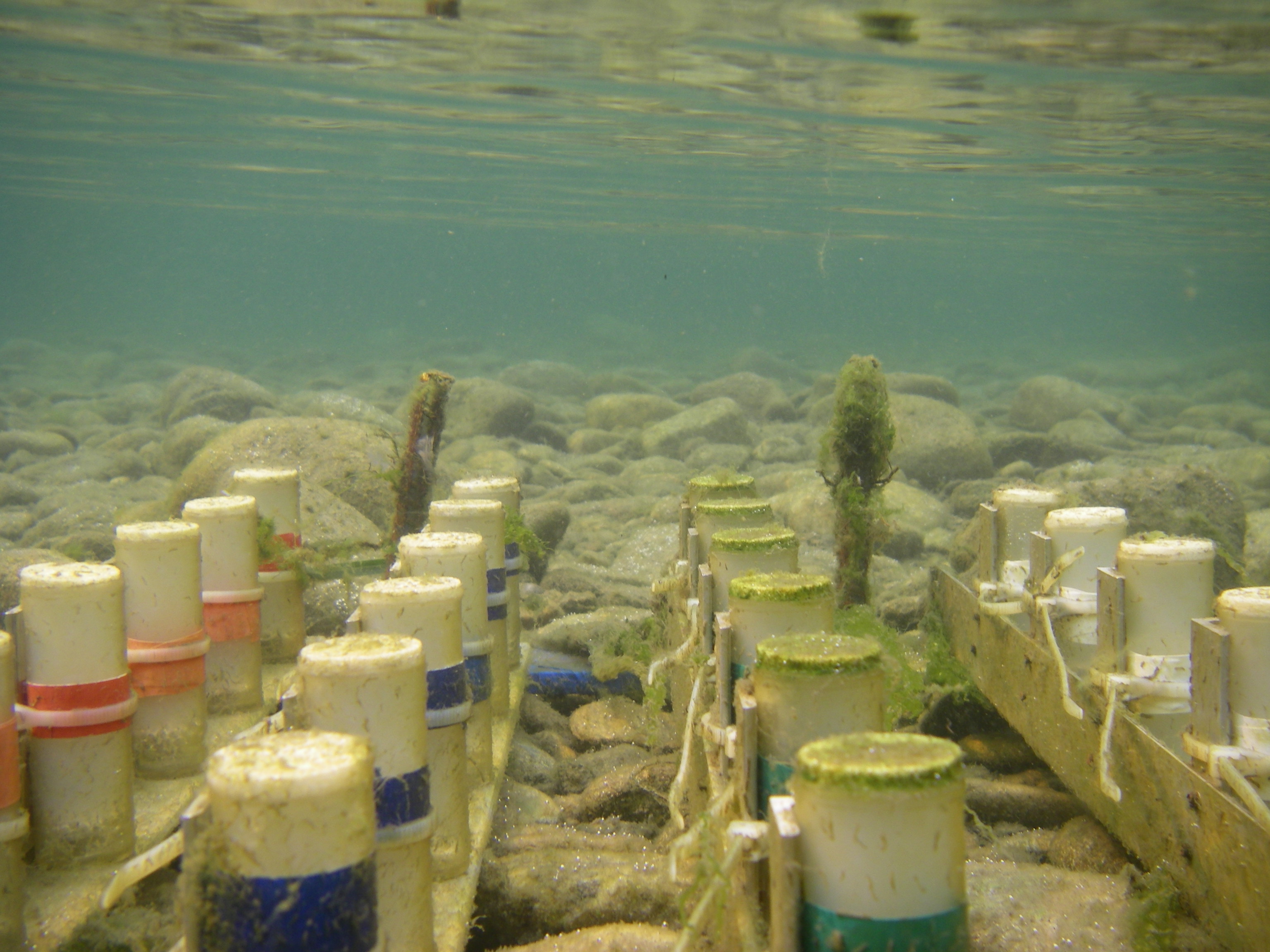

Supplement: S1 Image — (JPG) [file pone.0252904.s005.JPG]
