# Supplementary material for: Nutrient limitation of algae and macrophytes in streams: Integrating laboratory bioassays, field experiments, and field data
Source: PLoS One. 2021 Jun 18;16(6):e0252904. doi: 10.1371/journal.pone.0252904 (PMC8213151; doi:10.1371/journal.pone.0252904)

A. Site 10, Big Cottonwood Creek near Oakley, Idaho, a runoff influenced reference stream

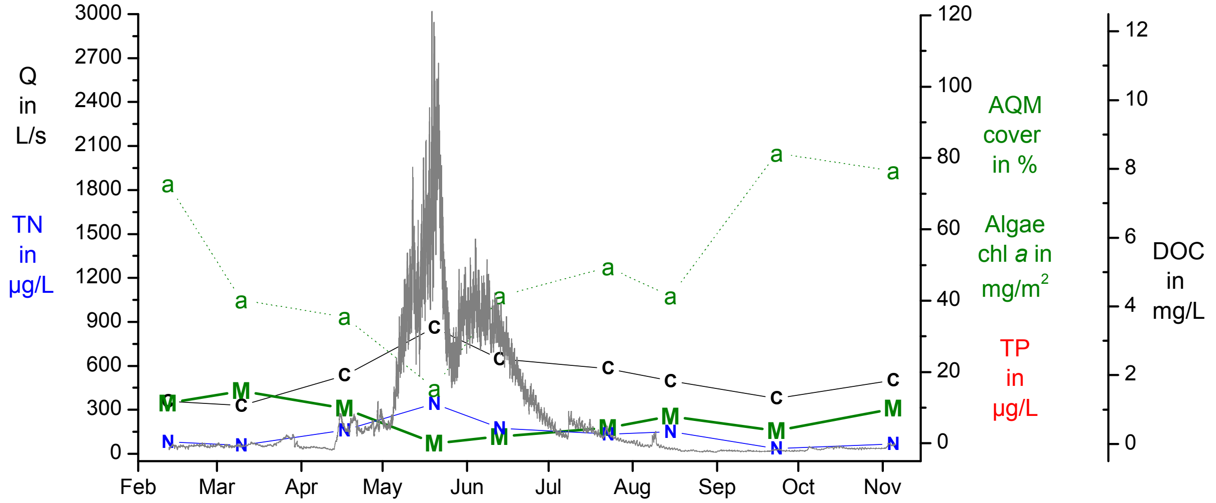

B. Site 26, Willow Creek near Bellevue, Idaho, a small spring-fed stream

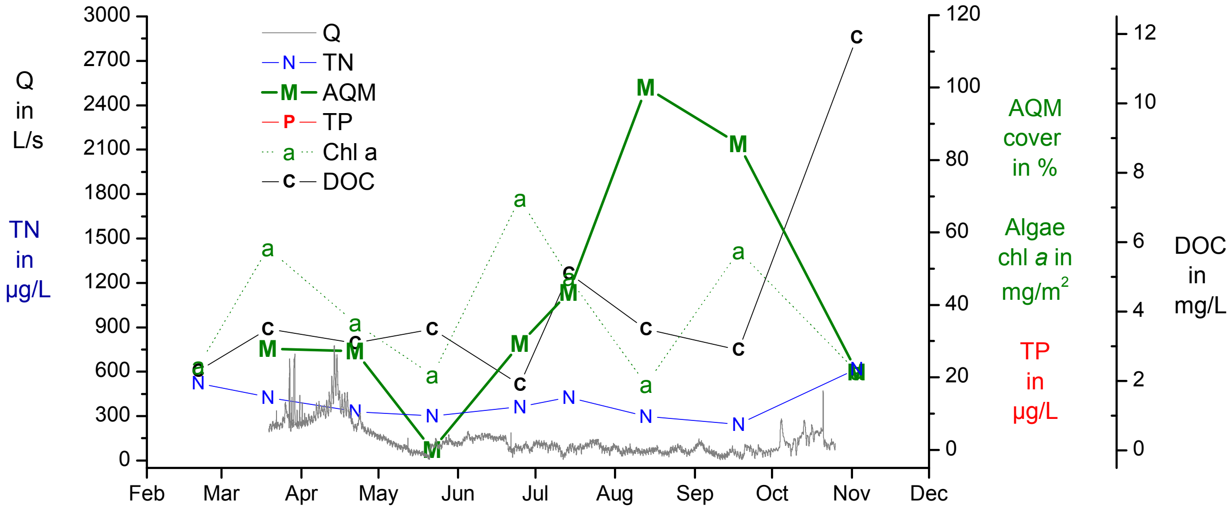

C. Site 24, Billingsley Creek near Hagerman, Idaho, a large spring-fed stream with high nutrients

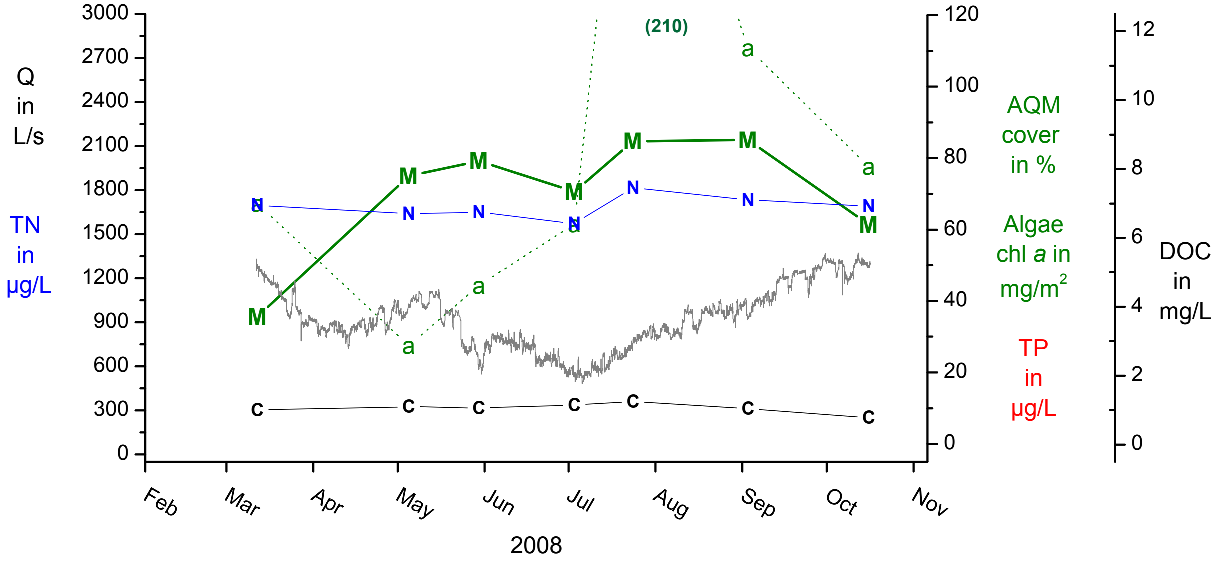

Site 25, Big Wood River near Bellevue Idaho, a runoff stream with low nutrient concentrations

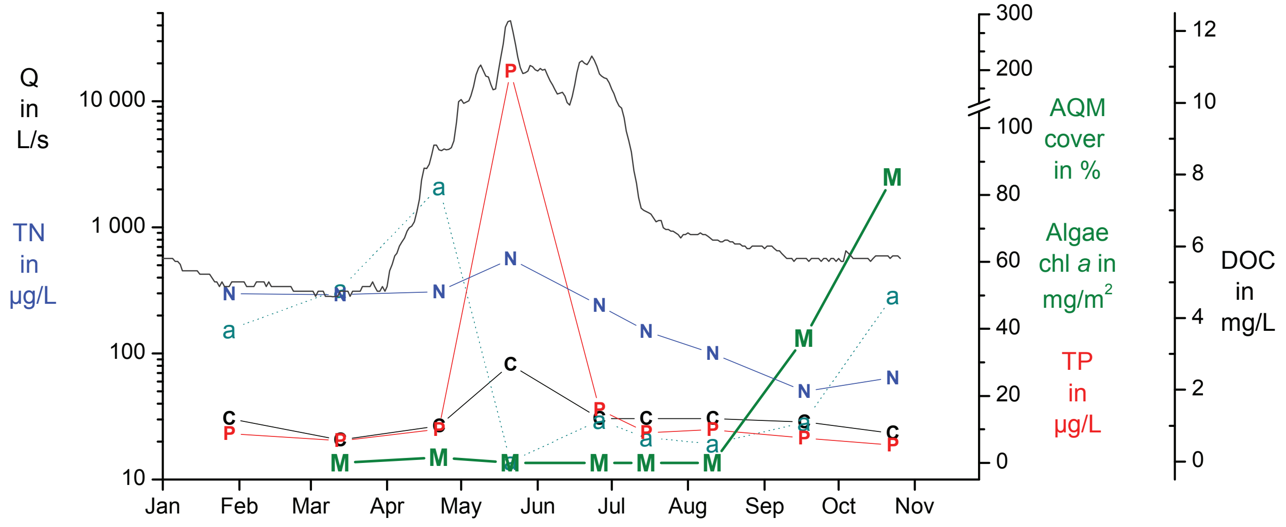

Site 28, Camas Creek near Blaine, Idaho, a runoff stream with high nitrogen concentrations

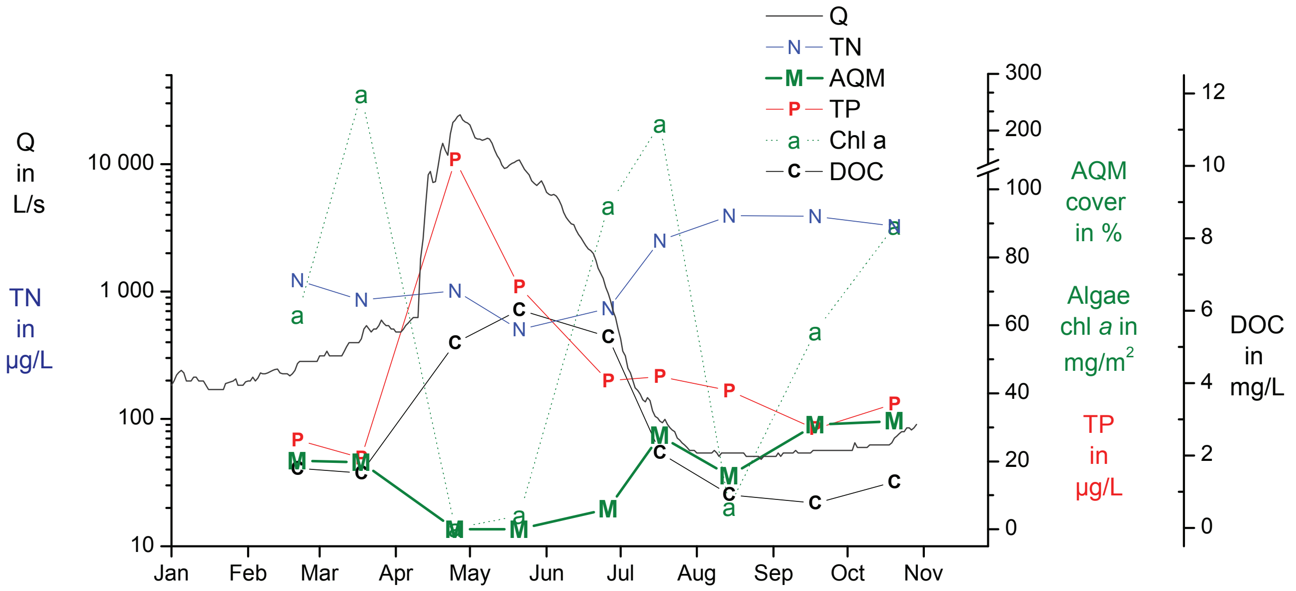

Site 29, Stalker Creek near Picabo, Idaho, a spring-fed stream with low phosphorus concentrations

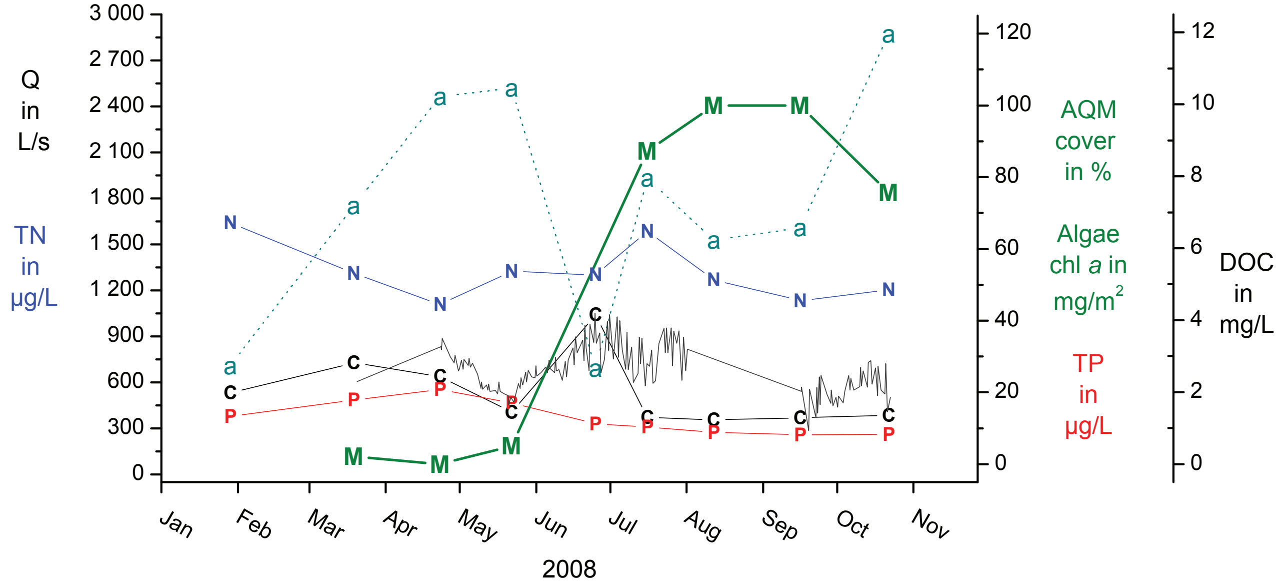

Supplement: S2 Fig — (PDF) [file pone.0252904.s002.pdf]
