# Supplementary material for: Nutrient limitation of algae and macrophytes in streams: Integrating laboratory bioassays, field experiments, and field data
Source: PLoS One. 2021 Jun 18;16(6):e0252904. doi: 10.1371/journal.pone.0252904 (PMC8213151; doi:10.1371/journal.pone.0252904)
Supplement: S1 File — (PDF) [file pone.0252904.s003.pdf]

Supplemental information on sites where nutrient diffusing substrates (NDS) were deployed

Contents

General information 1
Stalker Creek (stream with very low P and high N) 4
Big Cottonwood Creek (Pristine rangeland reference stream with low P and very-Low N) 5
Big Wood River (Stream with very low P and very low N) 6
Little Wood River (Stream with low P and very low N) 8
Goose Creek (Stream with intermediate P and intermediate N)) 9
Camas Creek (Stream with intermediate P and very high N) 11
Billingsley Creek (Stream with high P and high N) 12
References 13

General information

The nutrient diffusing substrate (NDS) racks were secured in locations with depth, velocities, and light that were reasonably representative for the study site and were incubated for 21 days. This incubation period was selected because it was just short of accrual periods that have been reported to lead to sloughing and loss of benthic algae biomass, short enough to avoid extensive invasion of the racks by grazing snails, and because of experience that the nutrients in the vials become depleted by about 28 days (Rugenski et al. 2008). The racks were checked on days 11 or 12 to remove debris and make sure that they were not in danger of coming out of the water as flows dropped in late summer. Ambient nutrient samples were collected from stream water before and after the deployments and once during the deployments. Extensive colonization occurred in all deployments. In some tests, the differences in growth were so pronounced that treatments were visually different (e.g., Figures 1 and 2). The test sites were selected to provide a range of nutrient concentrations and ratios, and in some cases the results gave empirical support to our hypotheses of how nutrients and benthic algae responded but in other instances results were unexpected.

Eight NDS experiments were attempted and seven were completed successfully. The unsuccessful test had drifting debris lodge on the rack which shaded parts of the N, P and NP treatments. Summaries of the successful experiments follow.

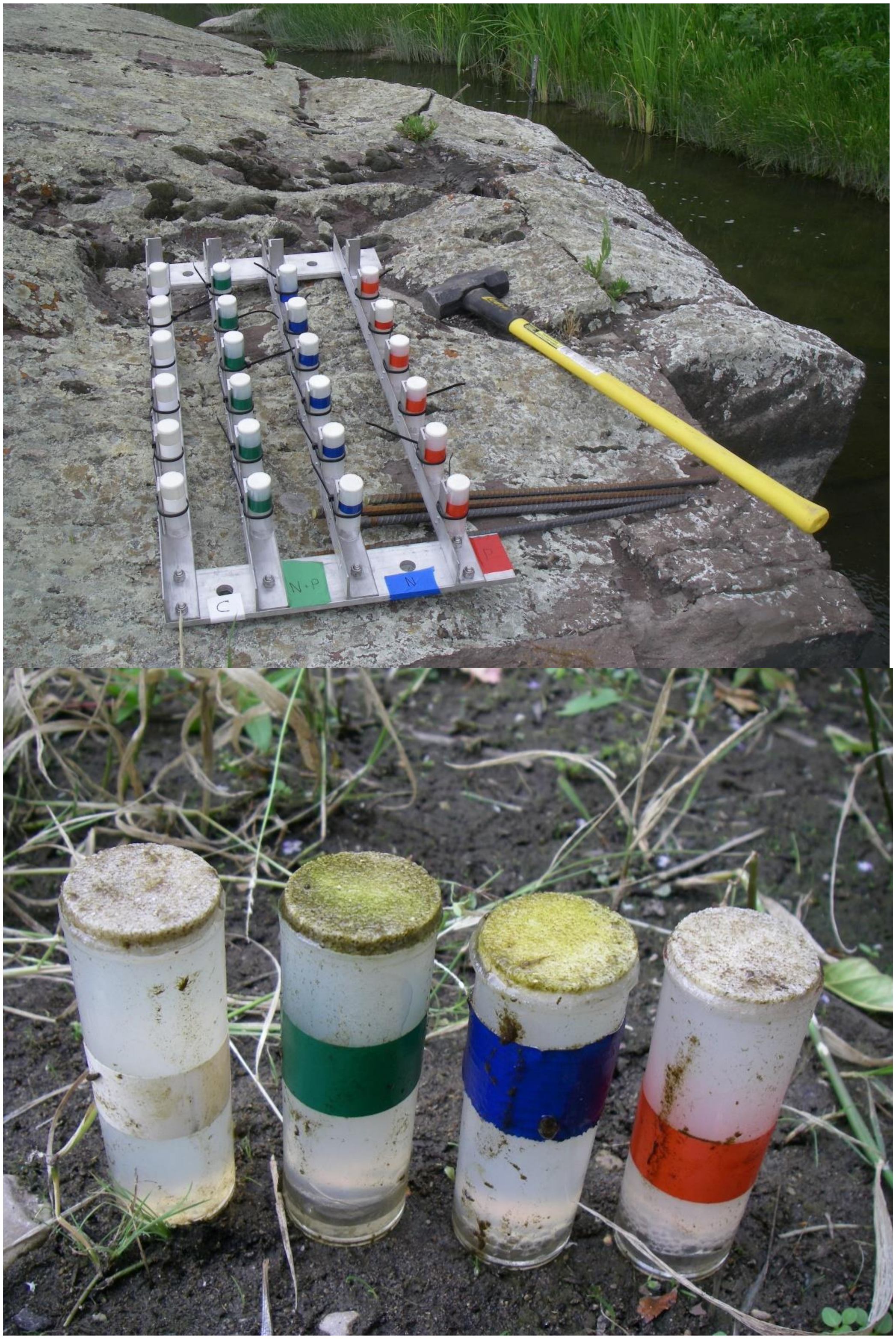

Figure 1. Rack of nutrient diffusing substrates (NDS) prior to deployment (left) and close-ups of the NDS vials showing differing periphyton accrual on the substrates following retrieval.

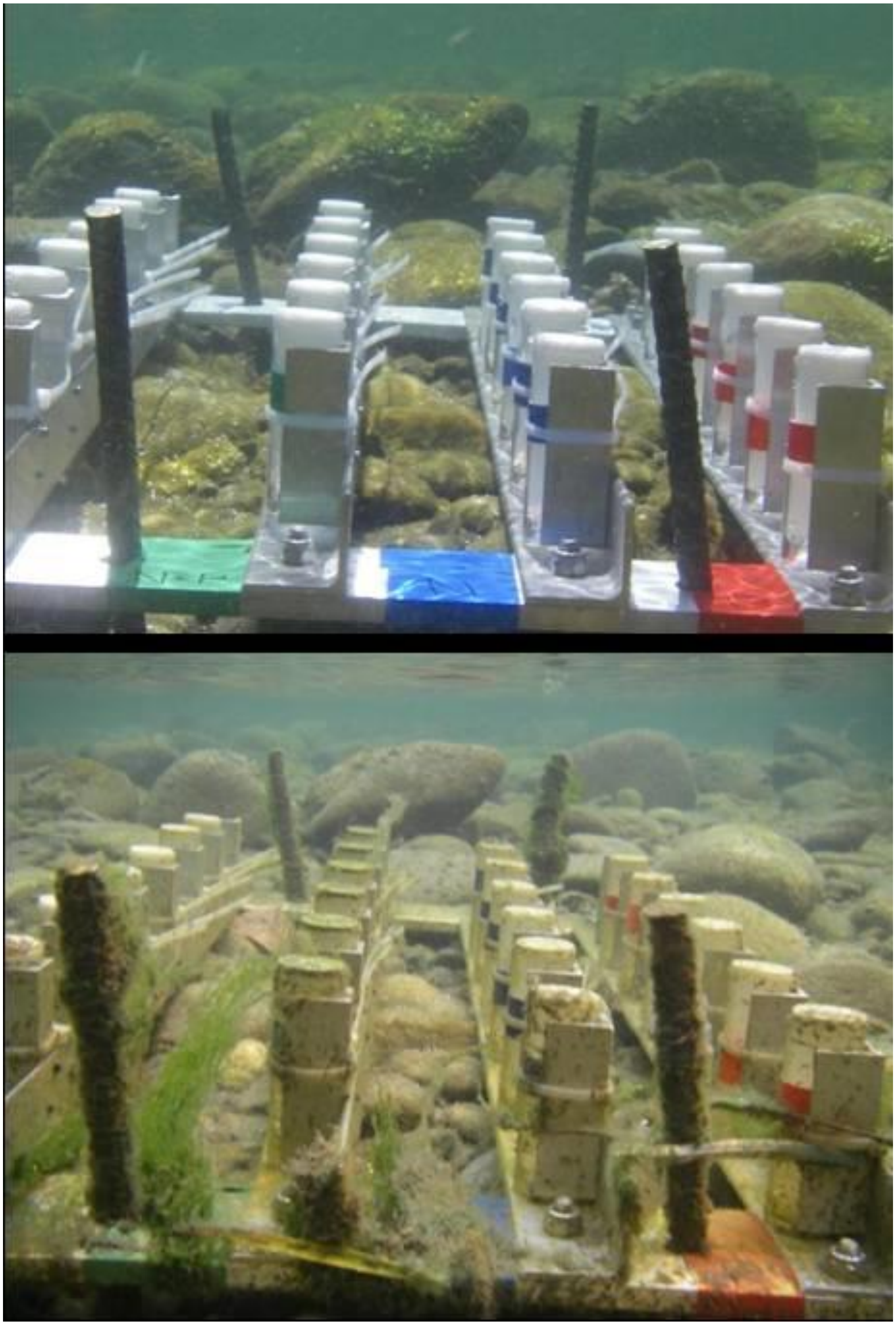

Figure 2. Matched, underwater views of NDS at deployment and after 21-days colonization in a N+P co-limited stream, the Big Wood River

Stalker Creek (stream with very low P and high N)

Stalker Creek was expected to be P limited based on the previous green algal growth test, the duckweed/periphyton test, and the N:P ratios greater than 300. The results of the NDS exposures were consistent with that expectation, showing primary P limitation (Figure 3).

Location details: USGS site number 13150200, geographic coordinates -114.18023, 43.31123260

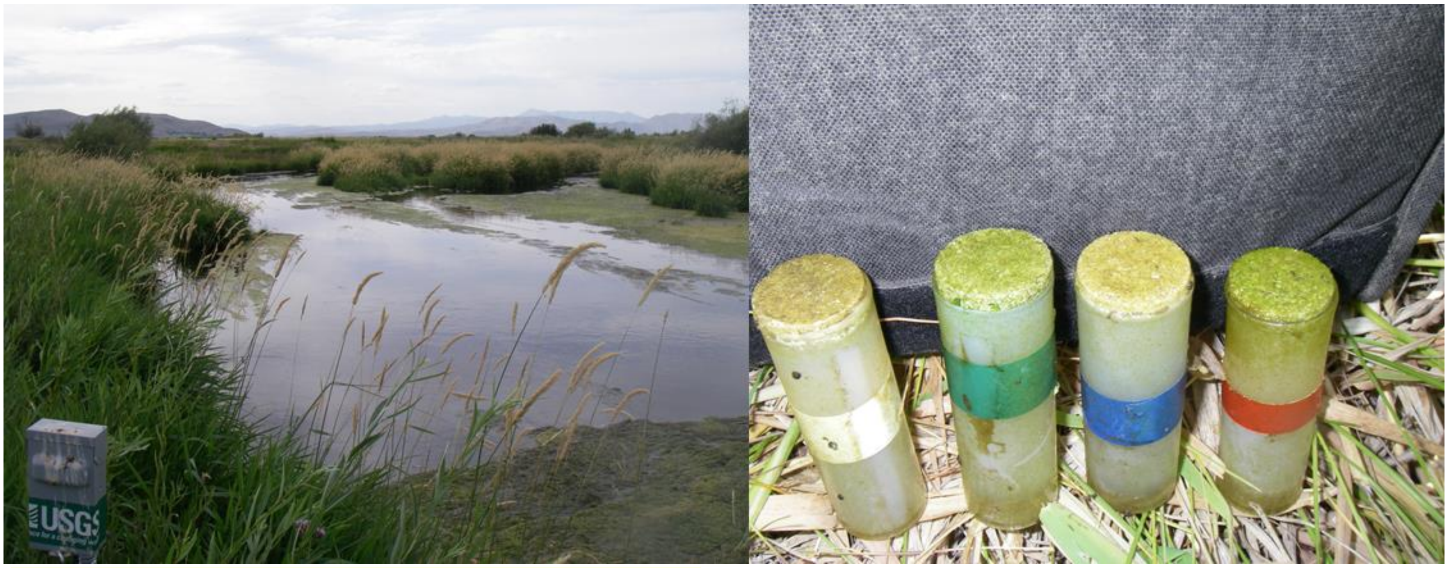

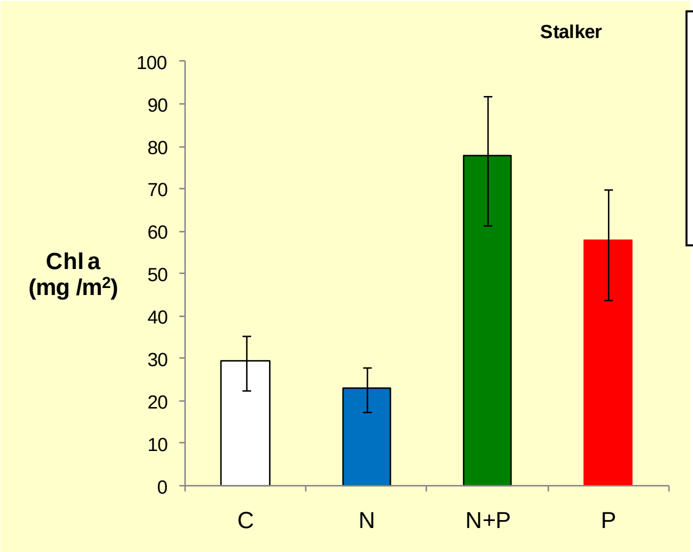

Ambient concentrations during deployment:
TP: 8 – 10 µg/L
TN: 1130 – 590 µg/L
N:P molar ratio: 340- 305

Conclusion: Primary P and secondary N limitation

Figure 3. Stalker Creek examples of differing algal accruals on the control or nutrient enriched substrates, ambient nutrient conditions and benthic chl *a* responses. Error bars show ±95th percentile confidence limits on the mean.

Big Cottonwood Creek (Pristine rangeland reference stream with low P and very-Low N)

Big Cottonwood Creek was expected to be N limited based on the previous green algal growth test, the duckweed/periphyton test, and ambient water N:P ratios less than 10 The results of the NDS exposures were not consistent with that expectation, showing N+P co-limitation. Phosphorous, added singly, suppressed overall benthic algae accrual (Figure 4).

Location details: USGS site number 13088510; geographic coordinates, -114.022944, 42.29371

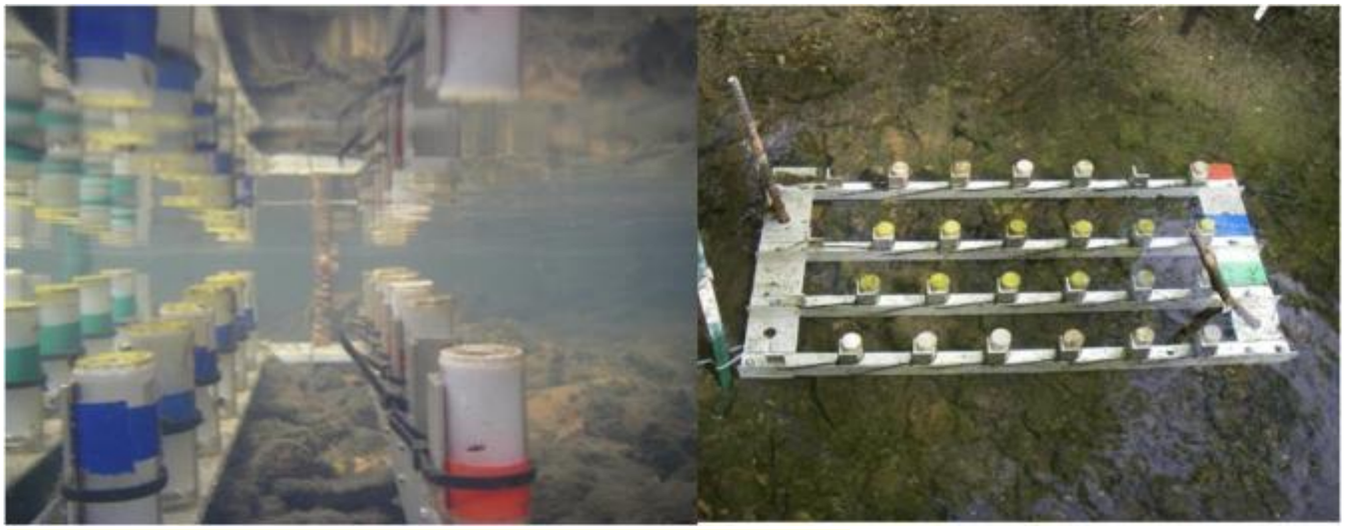

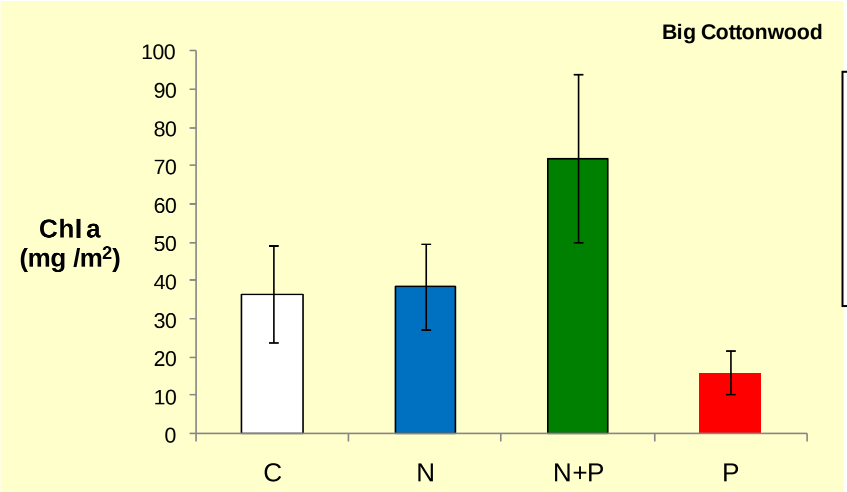

Ambient concentrations during deployment:
TP: 33 – 36 µg/L
TN: 35 – 135 µg/L
N:P molar ratio: 3 – 10

Conclusion: N and P co-limited

Figure 4. Big Cottonwood Creek showing the NDS rack just before removal, nutrient enriched substrates, ambient nutrient conditions and benthic chl *a* responses.

Big Wood River (Stream with very low P and very low N)

In the Big Wood River, ambient nutrient concentrations spanning the deployment were some of the lowest we measured anywhere in the study area with TP ranging only from 7 – 10 µg/L, and TN ranging from 50 – 150 µg/L. The N:P molar ratios for the deployment averaged 25, ranging from 15 to 37. These ratios predicting potential P nutrient limitation, using the >20:1 rule of thumb for potential P limitation. Instead, the actual nutrient limitation was strongly N and P co-limited. Benthic algae accrual was again lowest on the P amended substrates (Figure 5).

Location details: USGS site number 13140800; geographic coordinates, -114.321811, 43.326542

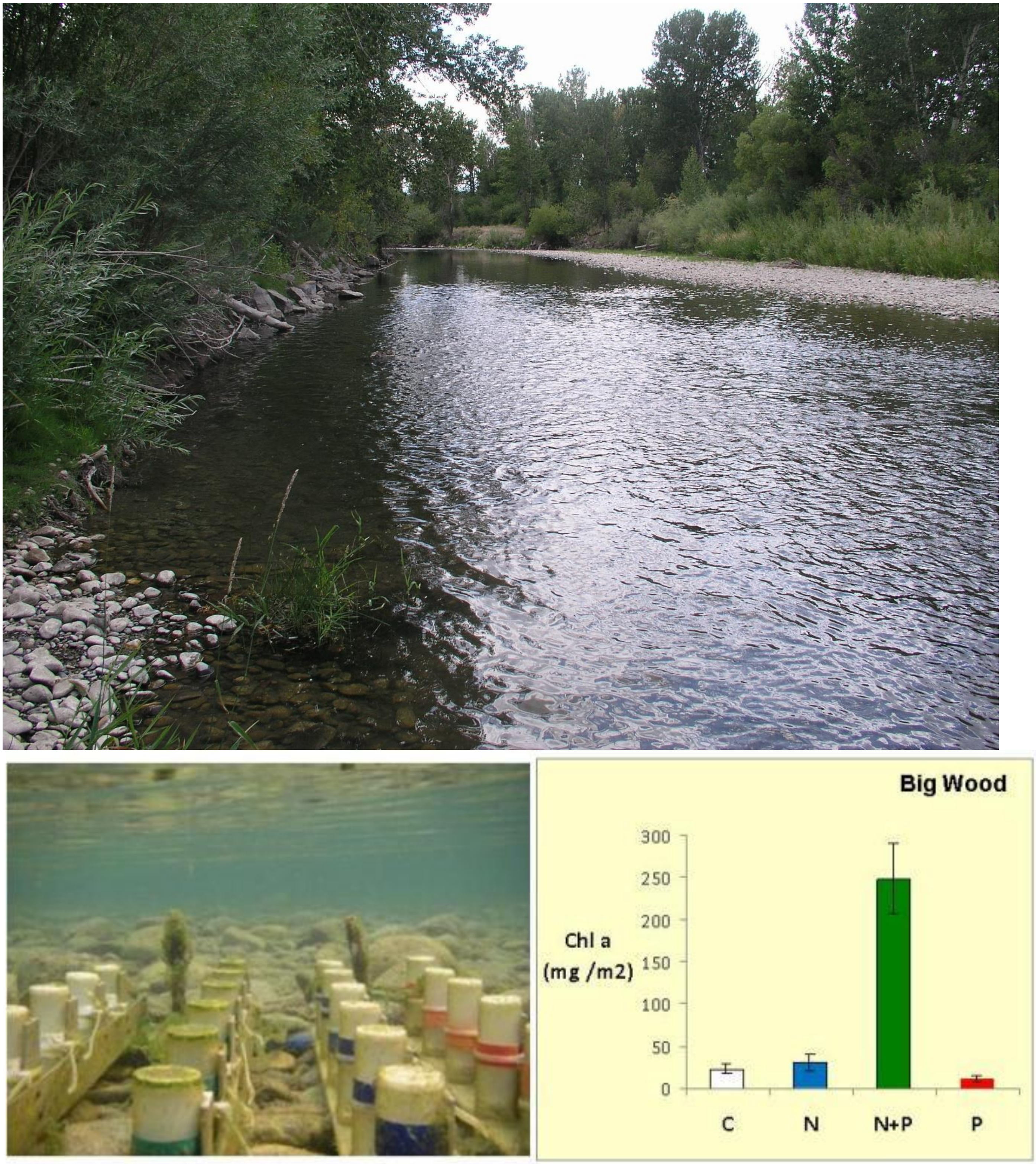

Figure 5. Periphyton accrual and chlorophyll (a) densities on unamended control substrates (C), nitrogen amended substrates (N), nitrogen and phosphorous amended substrates (N+P), and phosphorous amended substrates: Big Wood River.

Little Wood River (Stream with low P and very low N)

The conditions and algal responses in the Little Wood River make an interesting contrast with those of the Big Wood River because the ambient N concentrations in the Little Wood River study site were almost as low as those in the Big Wood River, but TP concentrations in the Little Wood were almost twice those measured in the Big Wood River. Nutrient N:P ratios averaged about 18 for the deployment, ranging from 14 to 20, which are equivocal for predicting potential nutrient limitation. The actual nutrient limitation was strongly N limited, again with the lowest algae accrual on the P amended substrates.

Location details: USGS site number 13147900; geographic coordinates, -114.057443, 43.493072

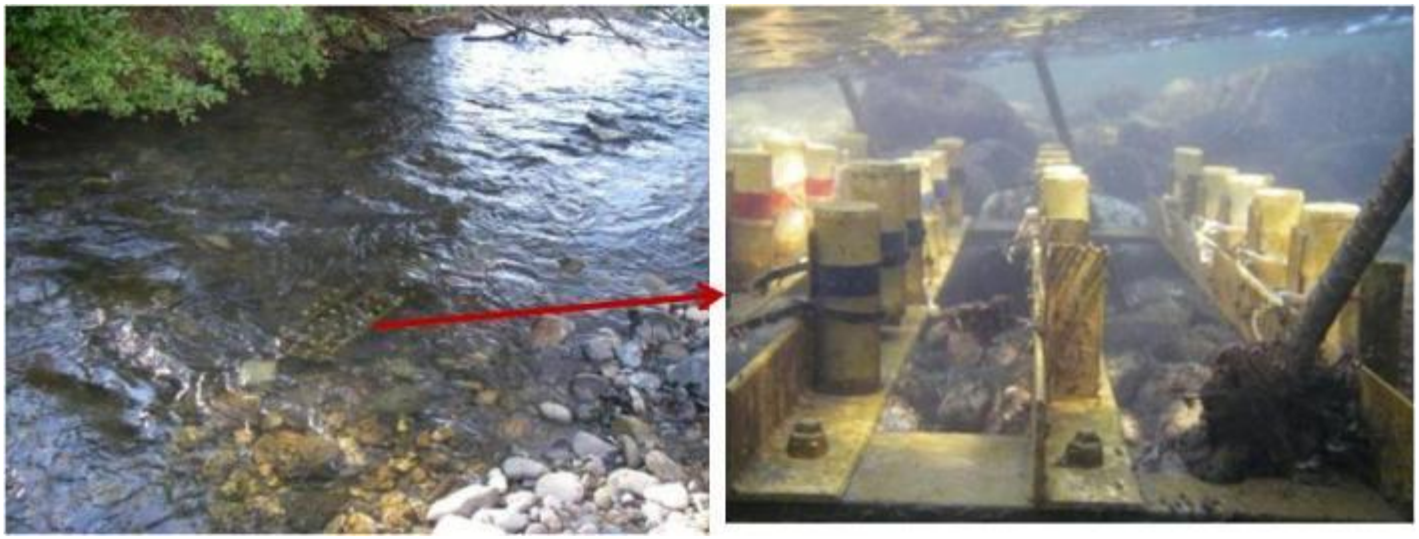

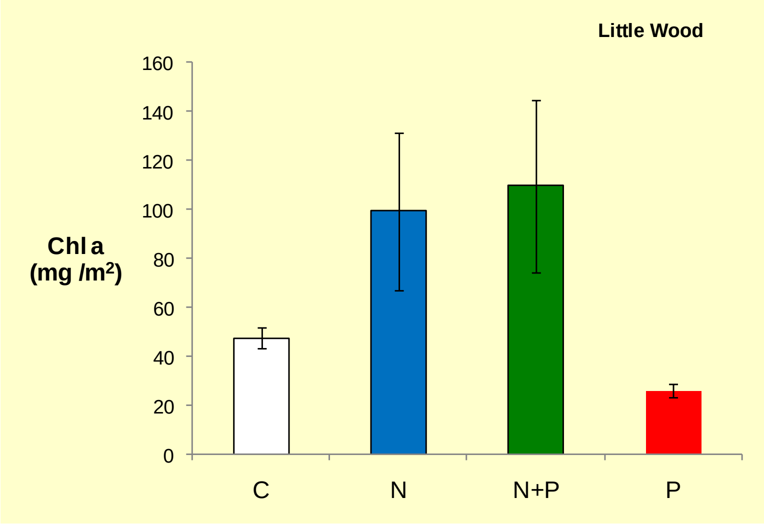

Ambient concentrations during deployment:
TP: 10 – 14 µg/L
TN: 93 – 118 µg/L
N:P molar ratio: 14 – 20

Preliminary Conclusion: N limited

Figure 6. Nutrient diffusing substrate exposures in the Little Wood River (top) and periphyton chlorophyll (a) accrual on the different nutrient substrate treatments (bottom)

Goose Creek (Stream with intermediate P and intermediate N))

The streams studied and pictured in Figures 1-6 could all be considered reference streams at the locations sampled, located within watersheds with light anthropogenic disturbance. In contrast, Goose Creek a rangeland stream located near the corner of Idaho, Utah and Nevada probably reflects a moderately disturbed stream due to abundant use of the riparian corridor for cattle grazing and frequent cattle trails through the stream channel. The NDS and racks visibly accumulated fine sediments along with the algal accrual during the deployment; contrast the rack and appearance of the disturbed rock in the foreground (Figure 7, top left) with the appearance after 21 days (Figure 7, top right).

The TP concentrations measured in Goose Creek were similar to those of Big Cottonwood Creek, the nearest reference stream, but TN concentrations were over twice as high (main article). Because Big Cottonwood was N+P co-limited, with twice as much N but similar P available, the expectation was that because of the more abundant N, Goose Creek might be P limited or would also be N+P limited. This was not the case, rather the results showed primary N limitation, with both the P and N+P having lower ending benthic chl *a* density than the N-only addition (Figure 7, bottom).

Location details: USGS site number 13082500, geographic coordinates -113.93468, 42.12755

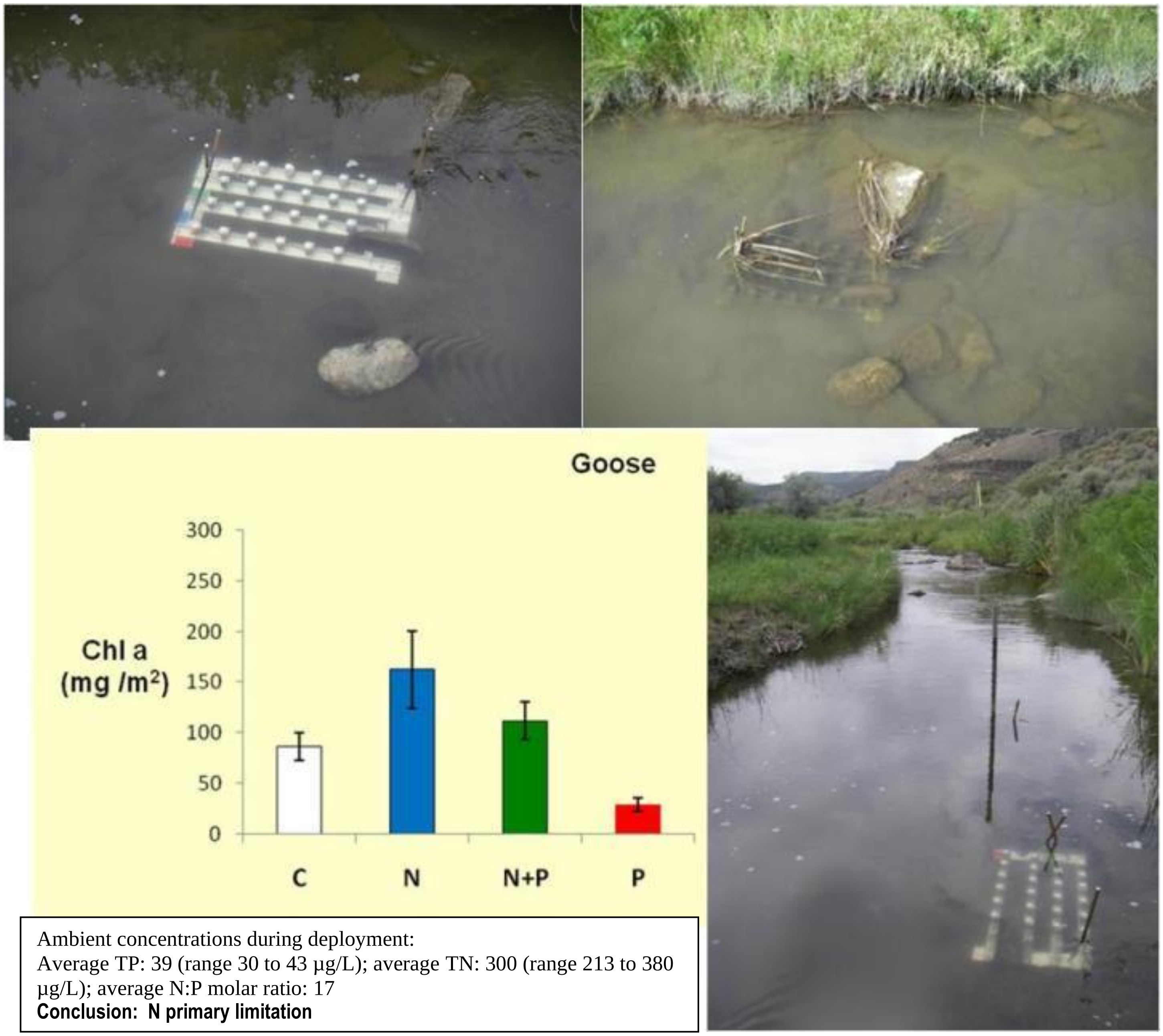

Figure 7. Nutrient-diffusing substrate rack at deployment and sediment buildup after 21-days deployment (top), and periphyton and chlorophyll a accrual: Goose Creek

Camas Creek (Stream with intermediate P and very high N)

Nutrient conditions in Camas Creek make an interesting contrast to the other streams because its TP concentrations are fairly typical for background among the rangeland sites we sampled, averaging 38 µg/L for the three sampling events spanning the deployments. However, TN concentrations were the highest we measured anywhere in the upper Snake River basin NEET study, averaging 3450 µg/L during the deployment. As expected, the NDS experiment showed primary P limitation in Camas Creek, but what was unexpected was the secondary N limitation (Figure 8). Because N was so abundant, the expectation was that adding more would have little effect.

Location details: USGS site number 13141500; geographic coordinates, -114.542225, 43.332762

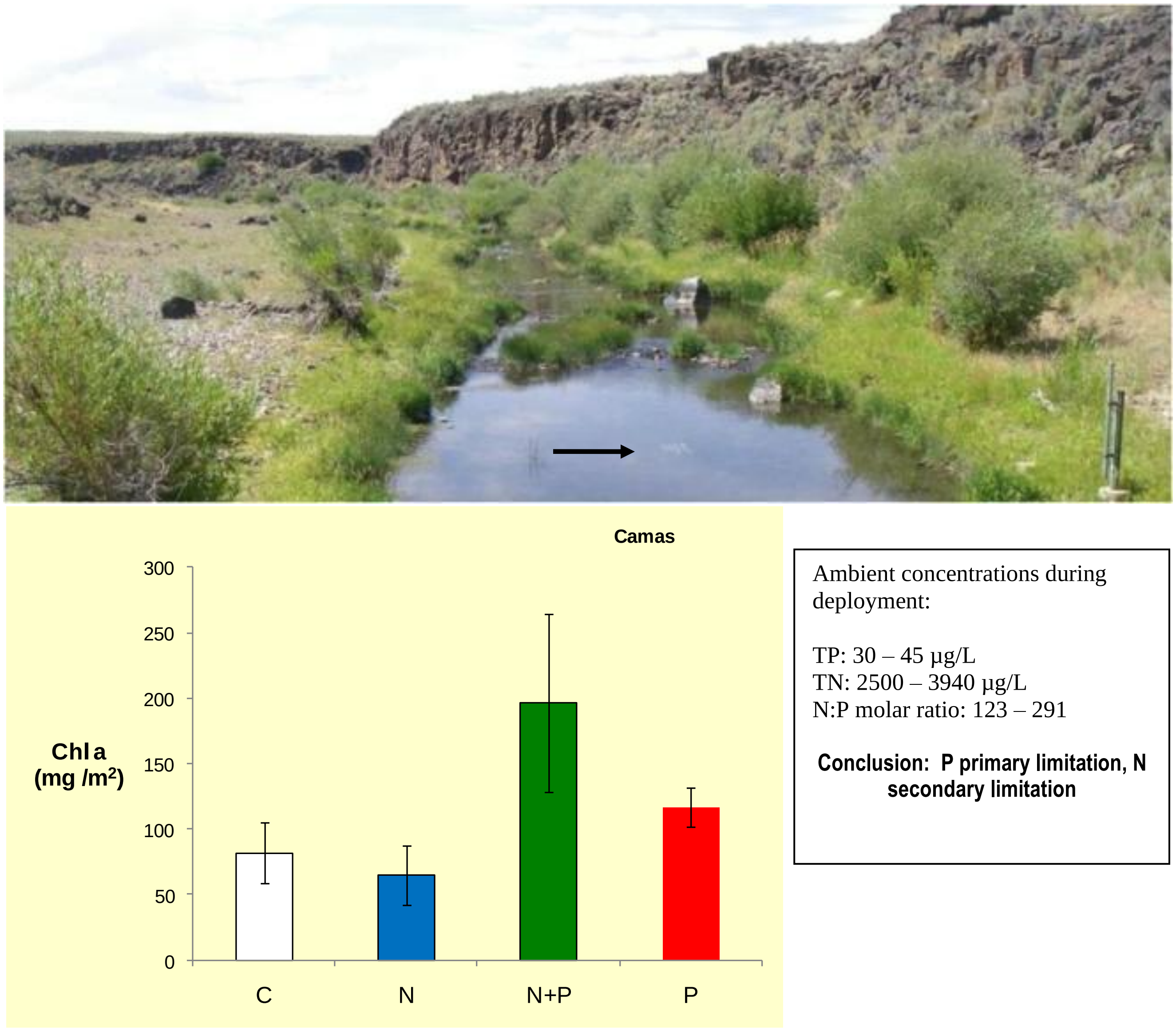

Figure 8. Nutrient diffusing substrate exposures in Camas Creek (top, by arrow) and periphyton chlorophyll (a) accrual on the different nutrient substrate treatments (bottom)

Sampling nutrients in Camas Creek during high and low flows and samples from a small spring in the canyon showed that the source of high N was from groundwater. The unusually high N may be of mostly natural origin, since most of the water in the stream during late summer came from sparsely populated subwatershed with only light agricultural practices (growing hay and cattle grazing) over a limited portion of the watershed. In other watersheds with volcanic geology, bedrock nitrogen has contributed to high nitrate concentrations in stream water (Holloway et al. 1998). Because we were investigating effects of nutrients in streams, not sources of nutrients in streams, we made only limited investigations of the reasons for the extremely high N, and only mention it because it was so anomalous.

Billingsley Creek (Stream with high P and high N)

The seventh and final nutrient limitation experiment discussed here was conducted in an enriched spring-fed stream in which both TP and TN are substantially elevated above natural background conditions. No nutrient limitation of benthic algae was apparent, with similar algae accruals on control, N, and P amended substrates. This was the only test in which the N+P additions resulted in the suppression of benthic algae.

Location details: USGS site number 13134640; geographic coordinates, -114.865404, 42.792406

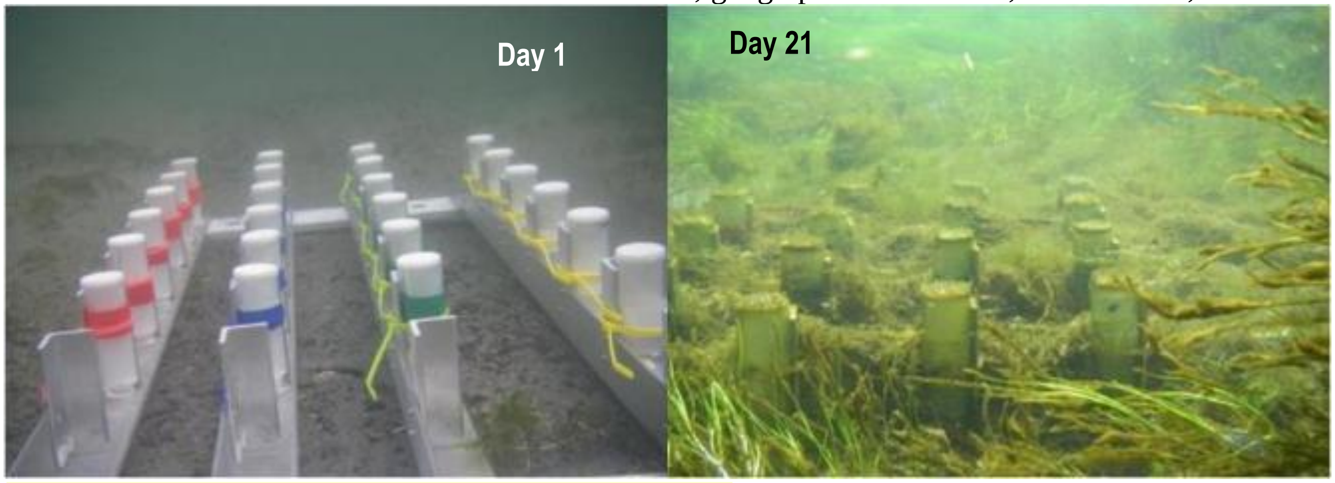

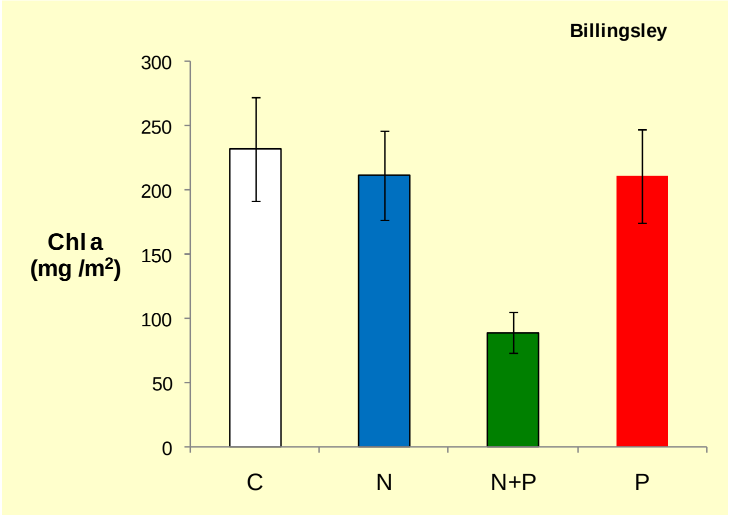

Ambient concentrations during deployment:

TP: 87 – 91 µg/L
TN: 1570 – 1820 µg/L
N:P molar ratio: 38 – 44

Conclusion: No nutrient limitation

Figure 9. Nutrient-diffusing substrate rack at deployment and plant growth after 21-days deployment (top), and periphyton and chlorophyll (a) accrual: Billingsley Creek

References

Holloway, R.A., R.A. Dahlgren, B. Hansen, and W.H. Casey. 1998. Contribution of bedrock nitrogen to high nitrate concentrations in stream water. *Nature*. 395: 785-788. *https://doi.org/doi:10.1038/27410*

Rugenski, A.T., A.M. Marcarelli, H.A. Bechtold, and R.S. Inouye. 2008. Effects of temperature and concentration on nutrient release rates from nutrient diffusing substrates. *Journal of the North American Benthological Society*. 27(1): 52–57. *https://doi.org/10.1899/07-046R1.1*
