# Supplementary material for: Nutrient limitation of algae and macrophytes in streams: Integrating laboratory bioassays, field experiments, and field data
Source: PLoS One. 2021 Jun 18;16(6):e0252904. doi: 10.1371/journal.pone.0252904 (PMC8213151; doi:10.1371/journal.pone.0252904)
Supplement: Attachment — Submitted filename: Responses to comments R2.docx [file pone.0252904.s007.docx]

# Responses to comments PONE-S-20-44903R1 “Nutrient limitation of algae and macrophytes in streams...”

Original comments in black text. Responses in blue text

## Reviewer 2

This manuscript presents the results from a study that used multiple laboratory- and field-based approaches to explore the boundaries of N and P limitation in a group of western US streams that lie along a gradient of anthropogenic nutrient enrichment. Nutrient limitation was assessed using a combination of low-concentration threshold and high-concentration saturation responses with bottle bioassays of a single algal taxon, laboratory growth trials of Lemna and its epiphytes, and deployments of nutrient-diffusing substrates in the field. I reviewed the original version of the manuscript. Overall, I find that the revision improved, even if the authors stuck to their guns regarding some of my criticism. I have both general and specific suggestions for its further improvement.

General Comments
More detail is needed about how the water samples were treated and analyzed. “Total” concentrations usually indicate that the sample is unfiltered and digested. Is this what the authors mean? If the authors actually mean “total dissolved” or “dissolved inorganic” then this should be made clear by laying it out in the methods and using the correct terminology.

We concur that this is an important distinction. We have gone through the text and clarified when we were referring to total P (unfiltered and digested) and total dissolved P (filtered and not digested), and likewise for N.

Specific Suggestions

1) Line 3: “…with water collected from nine streams in an agricultural…” Done

2) Line 4: “alga” Done

3) Line 5: “…test of periphyton were conducted with nutrient-diffusing…” Done

4) Line 8: Periphyton? Do the authors mean epiphyton? Changed to ‘epiphytic periphyton’

5) Line 11: “alga” Done

6) Line 14: Font size changes. Done

7) Line 19: “Our approach…” Done

8) Lines 16-18: Do the authors mean total dissolved concentrations here? Yes, but we are at our word limit. Specified in the text.

9) Line 44: Italicize r Done

10) Lines 80-84: If this paragraph is a justification for using Lemna then make it clearer. As is stands, this paragraph doesn’t make much sense. I still disagree with the authors about the need to put these methods-related sections in the introduction.

We revised this paragraph and it now hopefully clearer to set up our rationale on why we used a free-floating macrophyte in our testing. To us, explaining our rationale for doing the study we did is distinct from specific methods descriptions.

11) Line 117: “Streams are listed in order…” Done

12) Line 137: Missing period. Done

13) Line 149: “to the ambient water samples”? Reworded

14) Line 179: “algal species” Done

15) Line 198: Provide details of software. Done

16) How were these water samples filtered and analyzed? This is important in interpreting the results, as well as what the authors mean by “total” concentrations. Filtered and analyzed as total dissolved P and total dissolved N. Added a sentence at line 197 with more details.

17) Line 235: What kind of function was fit to the curve? Asymptotic. Annotated figure.

18) Line 250: Insert comma after “regression” Done

19) Line 262 and 264: “was removed” ‘Were removed’ seems appropriate here, as the sentences have plural subjects written: ‘90 percent of P and 80 percent of N were removed’

20) Line 329: “alga” Done

21) Line 332: “tests” Done

22) Line 375: Insert comma after “treatments” Done

23) Line 431: “instream” Done

24) Throughout: N-limited, P-limited, community-level, single-species, dual-nutrient, nutrient-saturated, nutrient-diffusing, rate-limited where modifying a noun. We’ve searched for and corrected all these and have taken another pass through to check the grammar for compound modifiers used as adjectives. While we don’t object to this criticism, we note that common usage is changing on this. For example, several references use “nutrient diffusing substrates” or “dual nutrient criteria.”
